# TIGIT reverses IFN-α-promoted Th1-like Tregs via in-sequence effects dependent on STAT4

**DOI:** 10.1186/s13075-023-03202-8

**Published:** 2023-11-17

**Authors:** Shihan Yu, Jia Gu, Rui Wang, Seunghyun Lee, Yu Shan, Jiakai Wang, Yini Sun, Xiaoxue Ma

**Affiliations:** 1 Department of Pediatrics, The First Hospital of China Medical University, No.155, Nanjingbei Street, Heping District, Shenyang, 110001 China; 2 Shanghai Children’s Medical Center Affiliated to Shanghai Jiao Tong University School of Medicine, Shanghai, China; 3 Xinhua Hospital Affiliated to Shanghai Jiao Tong University School of Medicine, Shanghai, China; 4 Department of Preventive Medicine, College of Medicine, Chung-Ang University, Seoul, Republic of Korea; 5 Department of Pediatrics, Shenyang Women’s and Children’s Hospital, Shenyang, China; 6 First Department of Internal Medicine, School of Medicine, University of Occupational and Environmental Health, Japan, Kitakyushu, Japan; 7 Department of Rheumatology and Immunology, The First Hospital of China Medical University, Shenyang, China; 8 Department of Critical Care Medicine, The First Hospital of China Medical University, Shenyang, China

**Keywords:** SLE, IFN-α, Th1-like Treg, TIGIT, Sequence effect

## Abstract

**Objectives:**

The induction direction of interferon (IFN)-α in T-cell phenotype and function varies depending on the activation state of the cell and the time of stimulation. To assess the effects of elevated IFN-α on regulatory T cells (Tregs) in systemic lupus erythematosus (SLE) patients, we investigated the differentiation of Th1-like Tregs under in-sequence and out-of-sequence conditions and the reversal effect of activating TIGIT on immune suppression.

**Methods:**

Phenotypes and activation levels of Tregs from SLE patients and healthy controls were analyzed using flow cytometry. In vitro culture conditions based on the sequence of TCR activation and IFN-α stimulation simulated in-sequence or out-of-sequence effects. CD4^+^T cells and Tregs were cultured under the above conditions with or without TIGIT agonist. Expression of related characteristic markers and phosphorylation levels of AKT, mTOR, and STATs were detected using flow cytometry and ELISA.

**Results:**

The frequency of Th1-like Tregs and activation levels of Tregs increased, but TIGIT expression in Tregs decreased in SLE patients. IFN-α promoted the conversation of Tregs to Th1-like Tregs while reducing immunosuppressive function under in-sequence conditions. The STAT4 pathway, but not the STAT1 pathway, was crucial for the IFN-α-mediated in-sequence effects. Reactivation of TIGIT reversed Th1 polarization of Tregs by suppressing AKT/mTOR and STAT4 signaling.

**Conclusions:**

Our findings suggest that IFN-α mediated in-sequence effects on Tregs may be responsible for the expansion of Th1-like Tregs in SLE. TIGIT can restore immune suppression damage in Tregs and represents a potential therapeutic target for SLE.

**Supplementary Information:**

The online version contains supplementary material available at 10.1186/s13075-023-03202-8.

## Introduction

CD4^+^T cells have high plasticity and can perform various functions by polarizing to different phenotypic subgroups in response to environmental factors [1]. Regulatory T cells (Tregs) are a special subgroup of CD4^+^T cells that develop from the thymus and peripheral blood [2]. Their regulatory immune functions and crucial role in maintaining immune homeostasis are well-established [3], but studies have shown that Tregs can be converted to effector/helper T (Th) cells. Although most Tregs retain high Forkhead box protein P3 (Foxp3) expression after adoptive transfer into a nonpathogenic setting, half of Tregs transferred into lymphogenic hosts begin producing interleukin (IL)-2 and interferon (IFN)-γ [4].

The expression of Th1 phenotype-related markers, such as C-X-C chemokine receptor 3 (CXCR3), T-box factor (T-bet), and C–C chemokine receptor type 5 is upregulated in Tregs in the presence of IL-12, IFN-γ, or IL-27, leading to the formation of Th1-like Tregs. These Tregs secrete high IFN-γ levels, significantly weakening immunosuppression [5, 6]. Changes in Treg stability blur the pro-inflammatory and anti-inflammatory cell lineages, weakening the suppressive function of Tregs [7], which provides the environmental conditions for the occurrence and exacerbation of autoimmune diseases. Accumulating research shows increased Th1-like Tregs numbers in patients with rheumatoid arthritis (RA), multiple sclerosis, and inflammatory bowel disease [8–10]. Up to now, there have been no reports on Th1-like Tregs in systemic lupus erythematosus (SLE). Our previous studies demonstrated that Th1-like phenotype polarization occurs through co-activation of the signal transducer and activator of transcription 1 (STAT1) and STAT4 pathways in patients with SLE [11]. Therefore, it is not difficult to imagine that Th1-like Tregs likely expand in SLE.

In addition, we observed that IFN-α pathologically increased in SLE patients [12]. IFN-α is widely recognized as a cytokine mediating Th1 polarization, which can activate almost all STAT pathways, especially those of STAT1 and STAT4 [13, 14]. However, elucidating the bidirectional functional characteristics of IFN-α is incomplete, and its induction effect on Treg cells is still unclear. The breakthrough point to assess this question lies in observing the relative timing sequence of IFN-α effects. IFN-α is highly sensitive to the sequential order of the activation of interferon-α receptor (IFNAR) and T cell receptor (TCR) signaling, which leads to two completely opposite effects on proliferation, apoptosis, and survival of T cells. When TCR stimulation slightly precedes that of IFN-α, IFN-α serves as a potent signal 3 cytokine to promote the proliferation and survival of T cells, known as “in-sequence signaling”. Conversely, delayed TCR engagement relative to IFNAR signaling induces an anti-proliferative and pro-apoptotic program called “out-of-sequence signaling” [15]. However, there are almost no studies on the sequential effects of IFN-α-mediated Treg phenotype conversion.

T-cell immunoglobulin and ITIM domains (TIGIT) are newly recognized co-inhibitory receptors detected on the surface of various lymphoid cells, which can improve the immunosuppressive function of Tregs and inhibit the activation of effector T cells [16]. Reduced TIGIT expression is observed in IL-12-induced IFN-γ-producing Th1-like Tregs [17]. In contrast, high TIGIT expression in Tregs is beneficial for inhibiting pro-inflammatory Th1 and Th17 cell responses [18]. In lupus mice, the TIGIT-immunoglobulin fusion protein can attenuate the immune response and inhibit the production of some autoantibodies [19]. TIGIT overexpression can alleviate the severity of RA by reducing IFN-γ and IL-17 expression and increasing the secretion of IL-10 [20]. The current study explores how IFN-α and TIGIT affect the differentiation direction and function of Tregs to elucidate the potential mechanisms of Th1-like Treg cells involved in SLE pathogenesis.

## Methods

### Patients

The clinical features of 33 SLE patients and 15 age- and gender-matched healthy donors (HDs) free of autoimmune and infectious diseases enrolled for measuring CXCR3^+^FOXP3^+^Tregs and TIGIT expression are shown in Supplementary Table S1. The clinical features of 16 SLE patients and 10 HDs enrolled for detecting T-bet^+^FOXP3^+^Tregs are presented in Supplementary Table S2. SLE patients were diagnosed by the 1997 American College of Rheumatology (ACR) criteria [21], the 2012 Systemic Lupus International Collaborating Clinics criteria [22], or the 2019 European League Against Rheumatism/ACR criteria [23]. Disease activity was measured by the SLEDAI score [24].

### Cell isolation and stimulation

Peripheral blood mononuclear cells (PBMCs) were isolated from SLE patients and HDs using Ficoll-Paque Plus (Cytiva, Uppsala, Sweden). Total CD4^+^T cells were purified using CD4^+^T cell Isolation Kit II (Miltenyi Biotec, Bergisch Gladbach, Germany). Tregs were purified with the CD4^+^CD25^+^ Regulatory T Cell Isolation Kit (Miltenyi Biotec). CD19^+^ B cells were purified using the B Cell Isolation Kit (Miltenyi Biotec). Cell purity was always > 85%. Cells were cultured in complete RPMI1640 medium (Hyclone) supplemented with 10% fetal calf serum in 96-well flat-bottomed plates (1.5 × 10^5^ cells/well). T cells were activated by TCR stimulation, and B cells were activated with lipopolysaccharide (LPS) (5 μg/mL) (Sigma, St Louis, MO, USA). Cytokines or antibodies used in the cultures are shown in Supplementary Table S3.

### Flow cytometry

Cells were stained with antibodies for 30–50 min. The antibodies are listed in Supplementary Table S3. For intracellular staining, cells were fixed for 50 min at 4 °C in Transcription Factor Buffer (BD Biosciences, San Jose, CA, USA) and then incubated in Perm/Wash Buffer I (BD Biosciences) for 50 min at 4 °C. For Phosflow, cells were incubated for 10 min with Phosflow Fix Buffer I and treated for 30 min at 4 °C with Perm Buffer III (BD Biosciences) before staining. Cells were examined by FACS Aria IIu flow cytometry, and data were analyzed using FlowJo 10.0 software (BD Biosciences).

### ELISA

IL-10 and IFN-γ levels in the medium from cultured cells were measured with ELISA kits (Absin, Shanghai, China). The concentrations of the cytokines were obtained from the mean fluorescence intensities (MFI) using MILLIPLEX software (Milliplex, Billerica, MA, USA).

### STAT knockdown and quantitative real-time PCR

CD4^+^T cells were incubated in Accell siRNA delivery medium and 1 μM STAT1, STAT4, or non-targeting control siRNA Accell SMART pool from Dharmacon (ABgene) for 48 h, then the cells were stimulated with TCR. Knockdown efficiency was assessed by quantitative real-time PCR. Total RNA was extracted from cells and purified using the RNeasy Mini Kit (Qiagen). cDNA was prepared using the High-Capacity RNA-to-cDNA kit (Applied BioSystems). Quantitative PCR was performed with the Sequence Detection System using site-specific primers and probes. The comparative threshold cycle method and an internal control were used to normalize the expression of the target genes.

### Statistical analysis

Data between different disease groups were compared using the Mann–Whitney’s *U* test. Comparisons between different groups were made using the Student* t*-test. Data were analyzed by GraphPad 8.0 and presented as the mean ± SEM. *P* < 0.05 was regarded as significant.

## Results

### Pathological expansion of Th1-like Treg cells and decreased immunosuppression in SLE patients

**Fig. 1:**
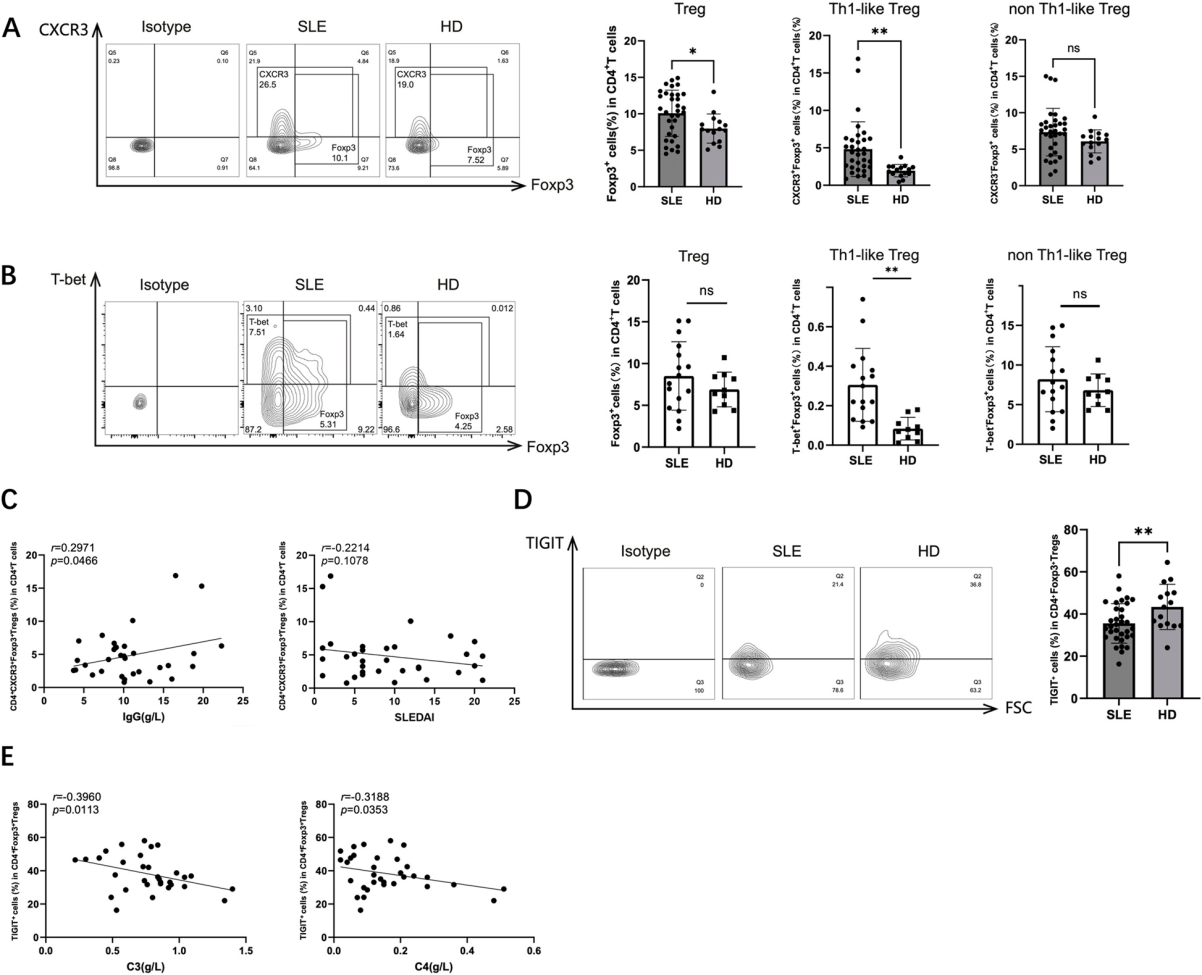
Th1-like Treg cells increased, accompanied by low expression of TIGIT in SLE patients. **A**, **C**–**E** PBMCs isolated from the peripheral blood of 33 SLE patients and 15 HDs shown in Supplementary Table S1 were analyzed using flow cytometry without incubation. **A** Frequencies of Foxp3^+^Tregs, CXCR3^+^Foxp3^+^Th1-like Tregs, and CXCR3^−^Foxp3^+^non-Th1-like Tregs in CD4^+^ T cells. **B** PBMCs isolated from the peripheral blood of 16 SLE patients and 10 HDs shown in Supplementary Table S2 were analyzed using flow cytometry without incubation. Representative flow cytometry plots and bar graphs showing the frequencies of Foxp3^+^Tregs, T-bet^+^Foxp3^+^Th1-like Tregs, and T-bet^−^Foxp3^+^non-Th1-like Tregs in CD4^+^ T cells. **C** Correlations of levels of IgG and SLEDAI with the proportion of Foxp3^+^Tregs from SLE patients. **D** The proportion of TIGIT^+^ cells in CD4^+^Foxp3^+^Tregs. **E** Correlations of levels of C3 and C4 with the proportion of TIGIT^+^CD4^+^Foxp3^+^Tregs from SLE patients. ns, not significant, *P < 0.05, **P < 0.01

First, we investigated the frequency of Th1-like Tregs in the peripheral blood of SLE patients and HDs. CD4^+^FOXP3^+^ total Tregs, CD4^+^CXCR3^+^FOXP3^+^Th1-like Tregs, and CD4^+^CXCR3^−^FOXP3^+^ non-Th1-like Tregs were identified using flow cytometry. Although the frequency of CD4^+^FOXP3^+^ total Tregs was unchanged in SLE patients, the frequency of CD4^+^CXCR3^+^FOXP3^+^Th1-like Tregs, but not non-Th1-like Tregs, significantly increased in SLE patients compared to the HDs (Fig. 1A). We also found that the frequency of CD4^+^T-bet^+^FOXP3^+^Th1-like Tregs, but not CD4^+^T-bet^−^FOXP3^+^ non-Th1-like Tregs, was significantly increased in SLE patients compared to the HDs (Fig. 1B). In addition, we measured clinical immune indicators and SLE disease activity index (SLEDAI) in the SLE patients to evaluate disease activity and severity. A positive correlation was observed between the frequency of Th1-like Tregs and total immunoglobulin G (IgG) levels (Fig. 1C), suggesting that Th1-like Tregs might contribute to the SLE autoimmune response.

We assessed TIGIT expression in the Tregs and found that its expression was decreased in the CD4^+^FOXP3^+^ total Tregs of SLE patients (Fig. 1D). We also evaluated the correlation between disease severity and TIGIT expression in Treg cells of SLE patients and found that complement 3 (C3) and C4 were negatively correlated with TIGIT expression in the Tregs (Fig. 1E), suggesting that a higher proportion of Tregs exhibited the Th1 phenotype in SLE patients compared to HDs, with low TIGIT expression and disrupted immune suppression function, which is related to the severity of SLE disease.

### IFN-α-mediated CD4^+^T cells are dependent on STAT1 signaling in out-of-sequence conditions and STAT4 signaling under in-sequence conditions

**Fig. 2:**
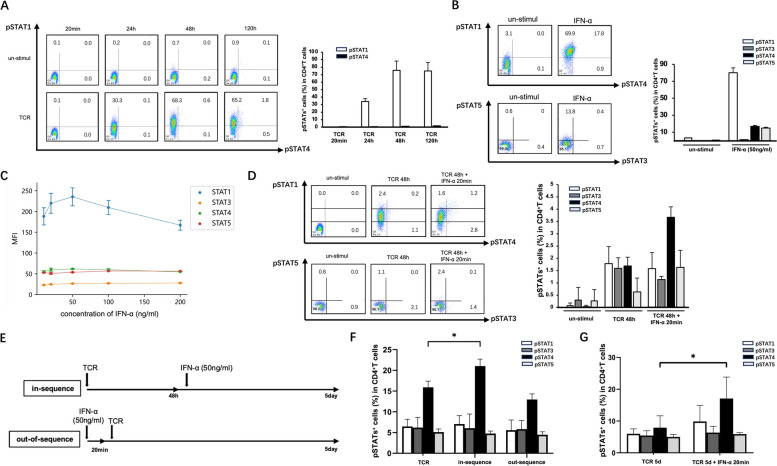
The different order in which IFN-α and TCR activate CD4^+^T cells can lead to the activation of different STAT pathways. CD4^+^T cells purified from PBMCs of HDs were stimulated at different time points, then analyzed using phosflow technology. **A** Percentages of pSTAT1^+^ cells and pSTAT4^+^ cells after TCR stimulated for 20 min, 24 h, 48 h, or 120 h. **B**, **C** Percentages and MFI of pSTAT1^+^ cells, STAT3^+^ cells, STAT4^+^ cells, and pSTAT5^+^ cells under the 20-min stimulation of IFN-α with specified concentration without TCR. **D** After 48 h of TCR-stimulation, percentages of pSTAT1^+^cells, STAT3^+^cells, STAT4^+^cells, and pSTAT5^+^cells were measured after further stimulation with or without IFN-α (20 ng/ml) for 20 min. **E** Schematic diagram of the setting in-sequence or out-of-sequence for culture conditions. **F** Percentages of pSTAT1^+^ cells, STAT3^+^ cells, STAT4^+^ cells, and pSTAT5^+^ cells after stimulation under TCR, in-sequence or out-of-sequence condition for 5 days. **G** After 5 days of TCR-stimulation, percentages of pSTAT1^+^cells, STAT3^+^cells, STAT4^+^cells, and pSTAT5.^+^cells were measured after further stimulation with or without IFN-α for 20 min. Data are mean ± SEM of three independent experiments using different donors. un-stimul, un-stimulation; min, minutes; h, hours; d, days. *P < 0.05

To clarify the effect of IFN-α on Treg differentiation, in vitro cultures were established, simulating the sequential order of the activation of IFNAR and TCR signaling. CD4^+^T cells were isolated from HD PBMCs and stimulated with anti-CD3 and anti-CD28 antibodies to activate TCR signaling. Time-gradient experiments indicated that the increasing STAT1 phosphorylation levels (pSTAT1) induced by TCR stimulation reached stable levels after 48 h, indicating full activation of TCR signaling in CD4^+^T cells (Fig. 2A). Moreover, pSTAT1, pSTAT3, pSTAT4, and pSTAT5 levels in CD4^+^T cells were all elevated to varying degrees after stimulation with exogenous IFN-α in the absence of TCR signaling, with the most significant increase observed with pSTAT1. IFN-α elevated and stabilized pSTAT1 levels within 20 min of exposure of CD4^+^T cells without TCR stimulation (Fig. 2B), which was concentration independent (Fig. 2C). In contrast, 48 h after complete activation of TCR, pSTAT4 levels, not pSTAT1 levels, in CD4^+^T cells were significantly enhanced by the subsequent addition of exogenous IFN-α (Fig. 2D).

Furthermore, we established out-of-sequence culture conditions by the addition of IFN-α to inactivated CD4^+^T cells 20 min before TCR stimulation and in-sequence culture conditions by adding IFN-α to CD4^+^T cells 48 h after TCR stimulation (Fig. 2E). To verify the long-term effects of the stimuli, CD4^+^ T cells were cultured for 5 days under only TCR activating, out-of-sequence, or in-sequence culture conditions. Under all conditions, pSTAT4 levels were relatively high after 5 days. Notably, pSTAT4 levels were significantly enhanced under in-sequence conditions compared to the other treatments (Fig. 2F). When cells cultured under only TCR stimulation for 5 days were subsequently stimulated by IFN-α for 20 min, pSTAT4, but not pSTAT1, was enhanced. These data suggested that the effect of IFN-α on CD4^+^ T cells was dependent on the STAT1 pathway under out-of-sequence conditions but dependent on the STAT4 pathway under in-sequence conditions.

### IFN-α promotes Th1-like Treg transformation under in-sequence conditions

**Fig. 3:**
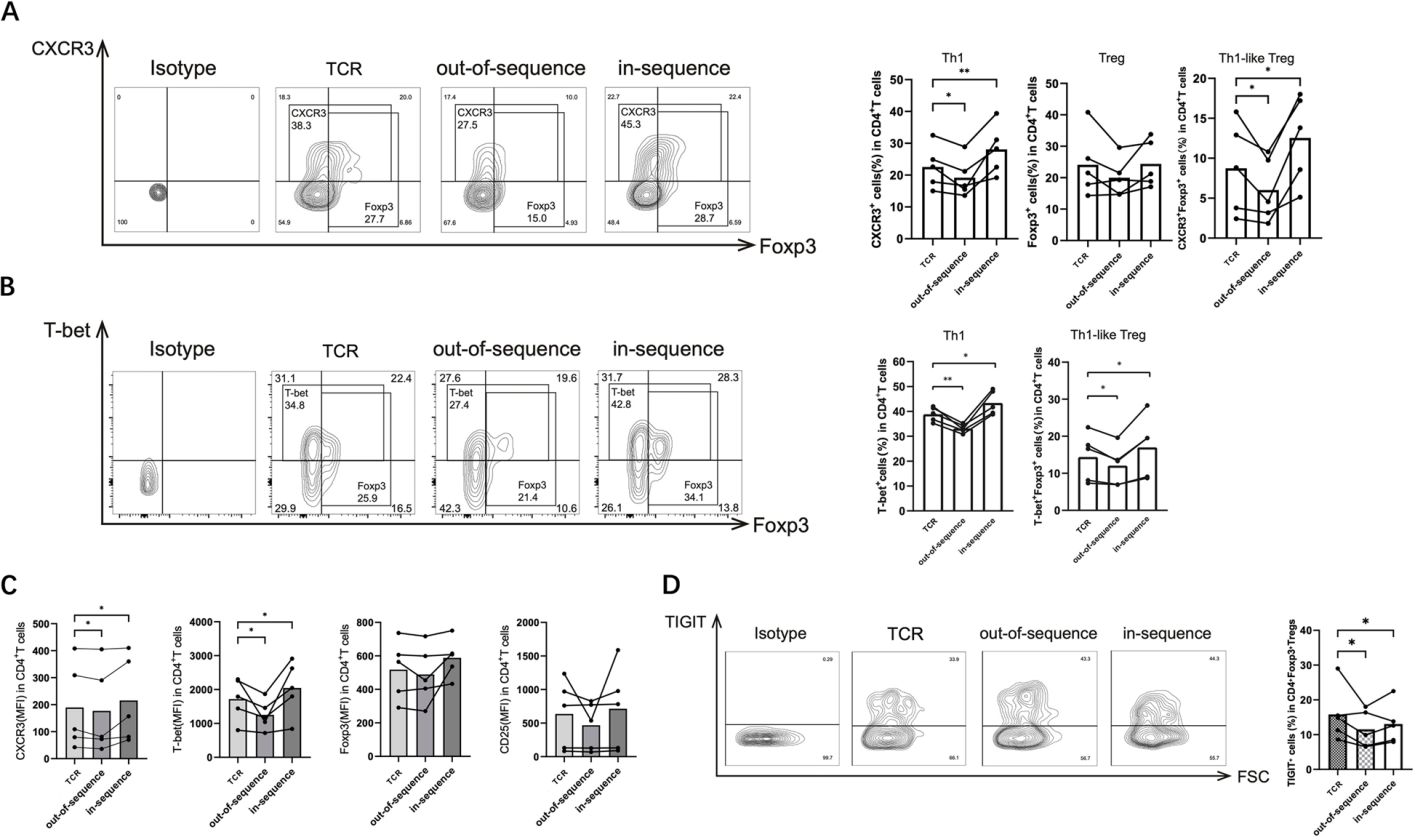
Th1-like Tregs were increased by IFN-α under in-sequence condition. CD4^+^CD25^+^Tregs purified from PBMCs of SLE patients were stimulated under TCR, out-of-sequence or in-sequence condition for 5 days, then analyzed using flow cytometry. **A** Percentages of CXCR3^+^Th1 cells, Foxp3^+^Tregs, and T-bet^+^Foxp3^+^Th1-like Tregs. **B** Percentages of T-bet^+^Th1 cells and T-bet^+^Foxp3^+^Th1-like Tregs. **C** Levels of T-bet, CXCR3, Foxp3, and CD25 expressed in MFI. **D** Percentages of TIGIT^+^ cells in Foxp3^+^Tregs. Data are mean ± SEM of five independent experiments using different donors. *P < 0.05, **P < 0.01

We investigated whether the differentiation of Th1-like Tregs was sensitive to the sequential effects of IFN-α. CD4^+^CD25^+^Tregs were purified and cultured under only TCR stimulating, out-of-sequence, or in-sequence conditions for 5 days. We found that the differentiation of CD4^+^CXCR3^+^Foxp3^+^ Th1-like Tregs increased under in-sequence conditions, whereas they decreased under out-of-sequence conditions (Fig. 3A). The same results were observed when defining Th1-like Tregs using T-bet and Foxp3 (Fig. 3B). CXCR3 and T-bet expression levels in the Tregs were increased by IFN-α under in-sequence conditions but decreased under out-of-sequence conditions, whereas Foxp3 and CD25 expression levels were not significantly affected (Fig. 3C). In addition, we found that TIGIT expression in the Tregs was reduced under in-sequence conditions (Fig. 3D). Together, our findings suggested that IFN-α could promote the transformation of Tregs into Th1-like Tregs by decreasing TIGIT expression under in-sequence conditions.

### IFN-α-induced Th1-like Tregs exhibit immunosuppressive dysfunction

**Fig. 4:**
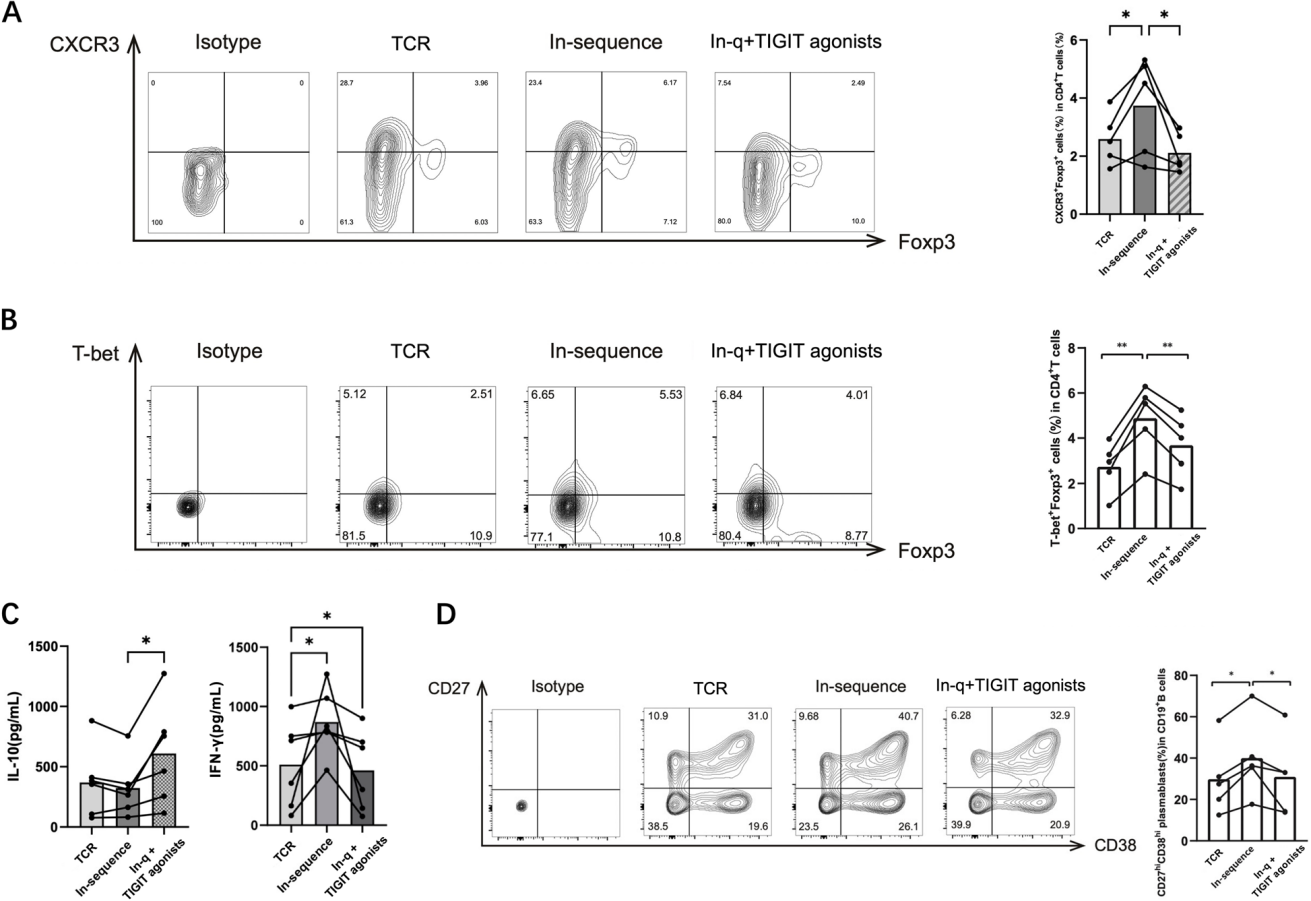
Impaired immunosuppressive function of Th1-like Tregs can be restored by reactivating TIGIT. CD4^+^CD25^+^Tregs purified from PBMCs of SLE patients were stimulated under TCR or in-sequence condition with or without TIGIT agonists for 5 days. **A** Percentages of CXCR3^+^Foxp3^+^Th1-like Tregs were analyzed using flow cytometry. **B** Percentages of T-bet^+^Foxp3^+^Th1-like Tregs were analyzed using flow cytometry. **C** Levels of IL-10 and IFN-γin cell culture supernatant were measured using ELISA. **D** CD25^+^Tregs were purified from cultured CD4^+^T cells, then co-cultured with CD19^+^B cells. Percentages of CD27^+^CD38.^+^plasmablasts were analyzed using flow cytometry. Data are mean ± SEM of five independent experiments using different donors. *P < 0.05, **P < 0.01

We also investigated whether IFN-α induction affected Treg-mediated immunosuppression. TIGIT agonist was used to activate TIGIT in Tregs. Interestingly, TIGIT activation reversed the conversion of Th1-like Tregs induced by IFN-α under in-sequence conditions (Fig. 4A, B). We further examined cytokine levels in the cell culture supernatant and found decreased levels of the suppressive cytokine IL-10 and increased secretion of the Th1-related proinflammatory cytokine IFN-γ under in-sequence conditions. TIGIT activation reversed those changes (Fig. 4C). To assess the suppressive effect of Treg cells on B-cell differentiation, IFN-α-stimulated Tregs were pretreated with TIGIT agonist and then co-cultured with B cells. TIGIT activation rescued the Th1-like Treg-mediated increase in plasmablasts (Fig. 4D). Therefore, TIGIT activation restored the immunosuppressive function of Th1-like Tregs reduced by IFN-α.

### STAT4 is essential for IFN-α-mediated differentiation of Th1-like Tregs

**Fig. 5:**
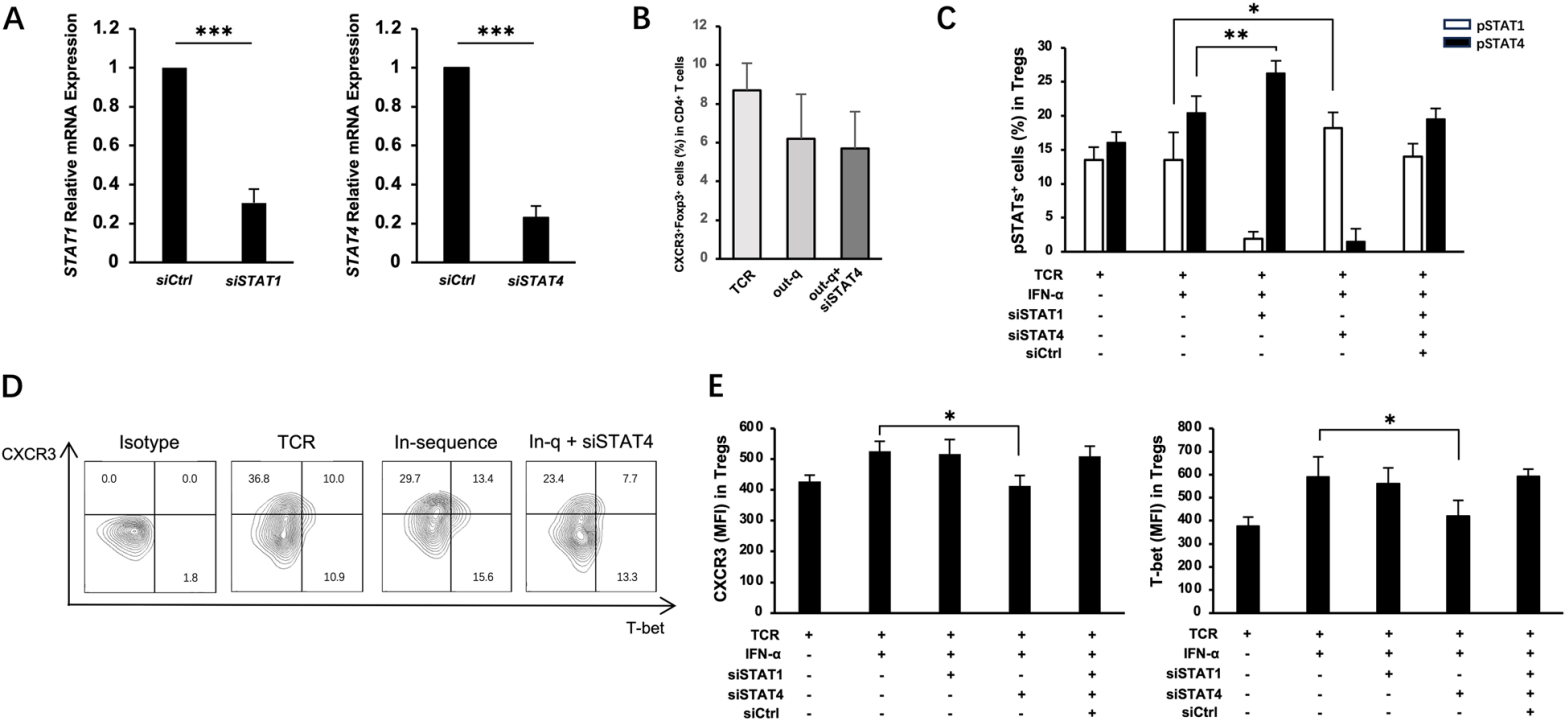
IFN-α improved Th1-like Tregs through STAT4 pathway in-sequence condition. CD4^+^ T cells were purified from PBMCs of HDs. **A** Relative messenger RNA expression of *STAT1* and *STAT4* in transfected CD4^+^ T cells was evaluated by quantitative PCR. **B** Knocking out STAT4 before exposing to specified conditions. Bar graphs showing the percentages of CXCR3^+^Foxp3^+^Th1-like Tregs analyzed using flow cytometry. **C**–**E** CD4^+^ T cells were activated by TCR before incubated under specified conditions and analyzed using flow cytometry. **C** Percentages of pSTAT1^+^ cells, and STAT4^+^ cells in Foxp3^+^Tregs. **D** Percentages of CXCR3^+^ cells and T-bet^+^ cells. **E** MFI of CXCR3 and T-bet in Foxp3^+^Tregs. Data are mean ± SEM of three independent experiments using different donors. *P < 0.05, **P < 0.01, **P < 0.001

We investigated whether the effect of IFN-α on Th1-like Treg differentiation was dependent on the STAT4 pathway for the in-sequence effect. CD4^+^T cells were pretreated with IFN-α and siRNA for 48 h before stimulating TCR signaling (i.e., out-of-sequence conditions). STAT1 and STAT4 mRNA expression levels were effectively silenced by siRNA (Fig. 5A). In the absence of STAT1, proliferation and differentiation of CD4^+^ T cells failed. STAT4 knockdown marginally aggravated the IFN-α inhibitory effect on the conversion of CXCR3^+^Foxp3^+^Th1-like Tregs under out-of-sequence conditions (Fig. 5B). Under in-sequence conditions, silencing of STAT1 increased pSTAT4, and STAT4 knockdown increased pSTAT1 (Fig. 5C). STAT4 knockdown, but not that of STAT1, abolished the expression of T-bet and CXCR3 induced in Foxp3^+^Tregs (Fig. 5D, E). These results indicated that IFN-α improved Th1-like Tregs under in-sequence conditions in a STAT4-dependent manner.

### Activating TIGIT restores the dysfunction of Tregs by specifically regulating the STAT4 pathway under in-sequence conditions

**Fig. 6:**
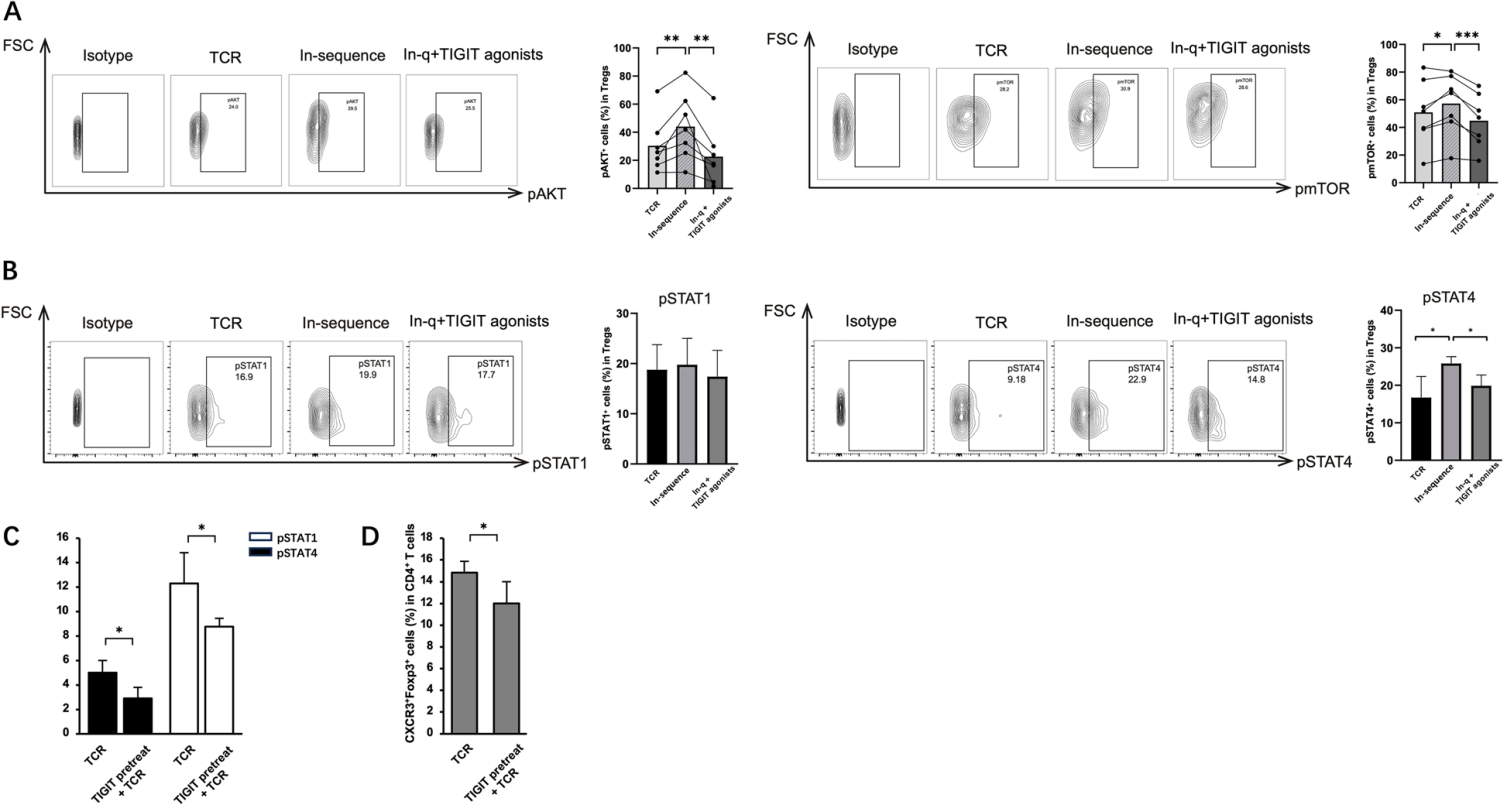
TIGIT can reverse Treg cell function and phenotype. CD4^+^ T cells purified from PBMCs of HDs were stimulated under specified conditions for 5 days and analyzed using flow cytometry. **A** Percentages of pAkt^+^ cells and mTOR ^+^ cells in Foxp3^+^Tregs. **B** Percentages of pSTAT1^+^ cells and STAT4^+^ cells in Foxp3^+^Tregs. **C**, **D** CD4^+^ T cells were pretreated using TIGIT agonists before exposing to TCR. **C** Percentages of pSTAT1^+^ cells, STAT4^+^ cells in Foxp3^+^Tregs. **D** Percentages of CXCR3^+^Foxp3^+^Th1-like Tregs. Data are mean ± SEM of seven independent experiments using different donors. *P < 0.05, **P < 0.01, **P < 0.001

Recent studies showed that TIGIT can repress the Akt/mTOR pathway [25]. We measured the phosphorylation levels of Akt (pAkt) and mTOR (pmTOR) in Tregs and found that both pAkt and pmTOR levels were elevated under in-sequence conditions, but the addition of the TIGIT agonist suppressed these elevated levels (Fig. 4A). Under in-sequence conditions, activating TIGIT significantly reduced pSTAT4 levels induced by IFN-α but did not affect the pSTAT1 and pSTAT4 levels caused by TCR activation (Fig. 6B). To verify the effect of TIGIT upstream of TCR, cells were pretreated by TIGIT agonists before TCR activation. The results showed that pre-activation of TIGIT reduced both pSTAT1 and pSTAT4 levels (Fig. 6C) and weakened the differentiation of Th1-like Tregs (Fig. 6D).

### The in-sequence effect of IFN-α dominates in SLE patients, thus exerting the effect of promoting Th1-like Treg depending on pSTAT4

**Fig. 7:**
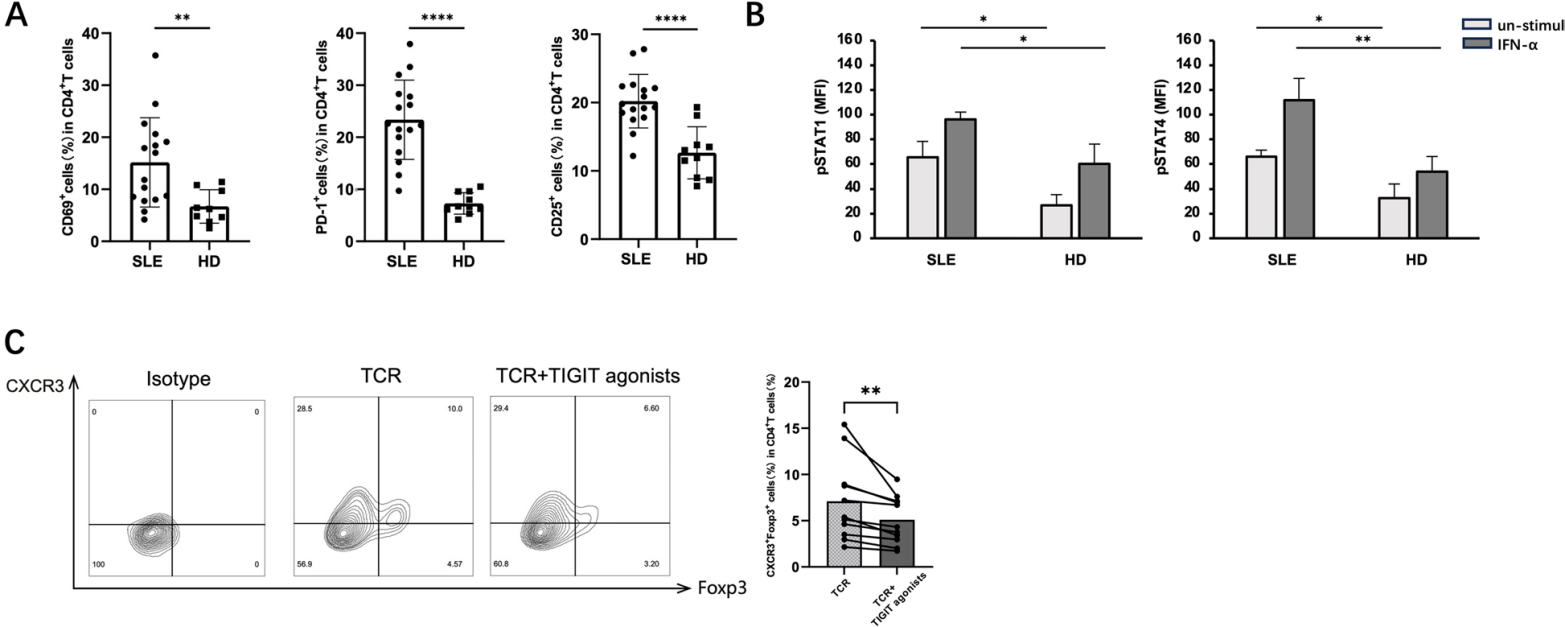
IFN-α mediated highly activated T cells following in-sequential effects in SLE. **A** PBMCs isolated from the peripheral blood of 16 SLE patients and 10 HDs shown in Supplementary Table S2 were analyzed using flow cytometry without incubation. Bar graphs showing the frequencies of CD69^+^ cells, PD-1^+^ cells, and CD25^+^ cells in CD4^+^ T cells. **B** Whole blood of patients with SLE was collected, stained directly or after the stimulation by IFN-α for 20 min. Bar graphs showing the MFI of pSTAT1 and pSTAT4 in Foxp3^+^Tregs analyzed using flow cytometry. **C** CD4^+^CD25^+^Tregs purified from ten PBMCs of SLE patients were stimulated under specified conditions for 5 days and analyzed using flow cytometry. Representative flow cytometry plots and bar graphs showing the frequencies of CXCR3^+^Foxp3^+^Th1-like Tregs in CD4^+^ T cells

Finally, we investigated whether IFN-α-mediated in-sequence effects caused the increase in Th1-like Tregs in SLE patients. The results showed that the expression levels of CD69, PD-1, and CD25 in the Tregs were all higher in SLE patients than in HDs (Fig. 7A). Without stimulation, the levels of pSTAT1 and pSTAT4 in the CD4^+^CD25^+^Tregs from SLE patients were higher than in HDs. IFN-α stimulation induced a more significant increase in pSTAT4 levels in the SLE patients (Fig. 7B). To explore the therapeutic effects of TIGIT on SLE, Tregs were treated with a TIGIT agonist and cultured under TCR stimulating conditions for 5 days. We found that the increase in the proportion of Th1-like Tregs was inhibited by the TIGIT agonist (Fig. 7C). In conclusion, our study showed that in SLE patients, Tregs were in a high TCR-activated state, and IFN-α could induce their differentiation into Th1-like Tregs through a STAT4-dependent in-sequence process, which could be reversed by TIGIT activation.

## Discussion

Elevated serum IFN-α levels in SLE patients play an important role in the pathogenesis of SLE [26]. In addition to anifrolumab, blocking or weakening the inflammatory function of downstream target cells regulated by IFN-α signaling is a potential therapeutic approach [27]. However, many studies have shown that there are conditional limitations on the role of IFN-α, especially related to inducing cell differentiation, which is controversial. Our results demonstrated that Treg differentiation is sensitive to the sequence of IFN-α and TCR signaling. Under in-sequence conditions, IFN-α promoted the polarization of TCR-activated Tregs towards the Th1-like phenotype, weakening their immunosuppressive function. The opposite occurred under out-of-sequence conditions.

The JAK/STAT signaling pathway is the primary pathway that explains all the biological effects of IFN-α. In T cells, IFN-α activates various STATs, which are influenced by the pathological environment [28–30]. In addition, genome-wide association studies (GWAS) identified multiple genetic loci associated with SLE, among which *TYK2*, *STAT1*, *STAT4*, and *IRF5* are key molecules directly involved in IFN-I signaling [31]. T cells carrying the STAT4 risk allele *rs7574865* are more sensitive to IFN-α, leading to increased pSTAT4 and secretion of IFN-γ [32]. Increasing STAT4 protein and gene levels in SLE patients are associated with the production of anti-dsDNA and lupus nephritis [33]. Our results showed that IFN-α promoted pSTAT4 under in-sequence conditions but pSTAT1 under out-of-sequence conditions. TCR signals may have a stronger affinity for STAT1 than IFN-α. For IFN-α, STAT1 could have a higher priority for activation than STAT4.

We found a significant increase in the proportion of Th1-like Tregs in SLE patients. At the same time, TIGIT expression in the SLE Tregs decreased, indicating that immune suppression was disrupted in SLE patients. The expression levels of CD69, PD-1, and CD25 in Tregs (i.e., indicators of T-cell activation [34–36]) were higher in SLE patients than in healthy individuals. The levels of pSTAT1 and pSTAT4 in Tregs from SLE patients were relatively higher than in Tregs from HDs before in vitro stimulation. After exposure to IFN-α, the pSTAT4 levels were elevated more significantly, indicating that the IFN-α-driving sequence effect dominated in SLE patients. Therefore, the IFN-α-mediated sequential effect in SLE could be the cause of increased Th1-like Tregs.

TIGIT is a promising new target for immunotherapy. TIGIT/CD155 engagement downregulates CD4^+^T-cell functions in SLE patients, indicating that TIGIT is a negative regulator of CD4^+^T-cell function in this disease [37]. TIGIT engagement induces the downregulation of the TCR-α chain and molecules that comprise the TCR complex, blocking productive T-cell activation [38]. In our study, pretreatment of inactive Tregs with a TIGIT agonist reduced TCR activation levels. Because TIGIT acts upstream in the TCR-induced signaling cascade, when TIGIT reactivation therapy is given to TCR-activated Tregs, the cellular program cannot be reversed. However, TIGIT reactivation can prevent TCR-activated Tregs from developing a Th1-like phenotype by affecting downstream signals [17, 25]. Our study demonstrated that the TIGIT agonist alleviated IFN-α-mediated inflammatory effects by reducing Akt/mTOR and STAT4 signaling under in-sequence conditions.

In summary, our study preliminarily explored how the time sensitivity of IFN-α affected Treg differentiation and function. SLE patients have a high TCR-activated state for Tregs, and in-sequence effects are the main response to IFN-α signals, leading to increased numbers of Th1-like Tregs and decreased immune suppression. Activating TIGIT can specifically reverse the immune tolerance caused by IFN-α-mediated in-sequence effects, making it a potentially effective treatment for SLE.

### Supplementary Information

**Additional file 1: Supplementary Table S1. **Demographic and Clinical Characteristics of Study Subjects 1. **Supplementary Table S2. **Demographic and Clinical Characteristics of Study Subjects 2. **Supplementary Table S3.** Antibodies used in the Study.

## Data Availability

All data generated or analyzed during this study are included in this published article and its supplementary information files.
