# Supplementary material for: TIGIT reverses IFN-α-promoted Th1-like Tregs via in-sequence effects dependent on STAT4
Source: Arthritis Res Ther. 2023 Nov 17;25:221. doi: 10.1186/s13075-023-03202-8 (PMC10655484; doi:10.1186/s13075-023-03202-8)
Supplement: Supplementary file 1 — Additional file 1: Supplementary Table S1. Demographic and Clinical Characteristics of Study Subjects 1. Supplementary Table S2. Demographic and Clinical Characteristics of Study Subjects 2. Supplementary Table S3. Antibodies used in the Study. [file 13075_2023_3202_MOESM1_ESM.docx]

**Supplementary Table S1.**

Demographic and Clinical Characteristics of Study Subjects 1

|  | SLE (n=33) | HD (n=15) | *P* |
| --- | --- | --- | --- |
| Age (year) | 40.7±13.7 | 36.1±13.4 | 0.280 |
| Females, n (%) | 30 (90.9%) | 14 (93.3%) | 0.958 |
| SLEDAI score | 9.6±6.3 |  |  |
| BILAG activity index | 15.8±7.6 |  |  |
| CRP (mg/L) | 13.1±18.8 |  |  |
| ESR (mmol/L) | 18.4±19.7 |  |  |
| IgA (g/L) | 3.6±5.9 |  |  |
| IgG (g/L) | 11.1±4.7 |  |  |
| IgM (g/L) | 1.0±1.1 |  |  |
| C3 (g/L) | 0.8±0.3 |  |  |
| C4 (g/L) | 0.2±0.1 |  |  |

Data are mean±SD or percentage of patients.

SLEDAI: Systemic Lupus Erythematosus Disease Activity Index; BILAG activity index: British Isles Lupus Assessment Group disease activity index; CRP: C-reactive protein; ESR: erythrocyte sedimentation rate; Ig: immunoglobulin; C: complement.

**Supplementary Table S2.**

Demographic and Clinical Characteristics of Study Subjects 2

|  | SLE (n=16) | HD (n=10) | *P* |
| --- | --- | --- | --- |
| Age (year) | 28.9±9.7 | 29.6±7.7 | 0.856 |
| Females, n (%) | 13 (81.25%) | 8 (80%) | 0.940 |
| CRP (mg/L) | 3.9±2.8 |  |  |
| ESR (mmol/L) | 15.8±10.2 |  |  |
| IgA (g/L) | 2.2±1.1 |  |  |
| IgG (g/L) | 10.2±3.1 |  |  |
| IgM (g/L) | 0.6±0.3 |  |  |
| C3 (g/L) | 0.9±0.3 |  |  |
| C4 (g/L) | 0.2±0.1 |  |  |

Data are mean±SD or percentage of patients.

SLEDAI: Systemic Lupus Erythematosus Disease Activity Index; BILAG activity index: British Isles Lupus Assessment Group disease activity index; CRP: C-reactive protein; ESR: erythrocyte sedimentation rate; Ig: immunoglobulin; C: complement.

**Supplementary Table S3.**

Antibodies used in the Study

| Antigen | Clone or Catalog | Company |
| --- | --- | --- |
| CD4-FITC | 555346 | BD Biosciences |
| CD4-APC-Cy7 | 357415 | Biolegend |
| CD3-APC-H7 | 560176 | BD Biosciences |
| CD25-APC | 555434 | BD Biosciences |
| PD-1-percp-Cy5.5 | 329914 | Biolegend |
| CD69-PE-Cy7 | 557745 | BD Biosciences |
| Foxp3-PE | 12-4776-41 | Invitrogen |
| T-bet-PE-Cy7 | 4B10 | Biolegend |
| CXCR3-BB700 | 1C6/CXCR3 | BD Biosciences |
| CD27-APC | 356409 | Biolegend |
| CD38-PE | 356603 | Biolegend |
| TIGIT-PE-Cy7 | 372713 | Biolegend |
| pSTAT1-AF488 | pY701 | BD Biosciences |
| pSTAT3-PE | 558557 | BD Biosciences |
| pSTAT4-APC | 17-9044-42 | Invitrogen |
| pSTAT5-PE-Cy7 | 560117 | BD Biosciences |
| pAKT-APC | 17-9715-41 | Invitrogen |
| pmTOR-PE-Cy7 | 25-9718-41 | Invitrogen |
